# Multi-objective optimization for minimize cutting force and power consumption in corn stalk chopping processes

**DOI:** 10.1016/j.heliyon.2024.e35373

**Published:** 2024-07-26

**Authors:** Kurre Prasanth Kumar Reddy, S. Ramesh Kumar, Boggarapu Nageswara Rao

**Affiliations:** Department of Mechanical Engineering, Koneru Lakshmaiah Education Foundation, Deemed to be University, Green Fields, Vaddeswaram, Guntur, 522302, India

**Keywords:** Chauvenet's criterion, Corn stalks, Approach angle, Feeding angle, Velocity

## Abstract

This study was on optimization of the cutting processes for corn stalks, which serve as significant sources of biomass and forage. The reduction of cutting force is crucial for enhancing energy efficiency. Cutting velocity rises, which can lead to significant power usage with minimal shear forces. Therefore, the objective is to minimize both the cutting force and power consumption. To address this multi-objective optimization problem, a modified Taguchi method was employed. Chauvenet's criterion was used to assess the validity of statistical scatter in repeated test data. The test samples had an 81 % moisture content. Through analysis of variance (ANOVA), the optimal chopping process parameters were identified as velocity of 4.4 m/s, approach angle of 30°, and feeding angle of 50°. The refined empirical relationships indicated that the cutting force ranges from 180.55 N to 393.37 N, while the cutting power ranges from 16.81 W to 44.05 W. In a single test, the output responses for the optimal parameter set were 26.32 W for power consumption and 242.60 N for cutting force, both are within the estimated range. The considerable scatter observed in repeated test data is likely due to variations in corn stalk thickness. These findings are valuable for the operation of chopping machines, ensuring minimal cutting force and power consumption when handling corn stalks residues.

## 1 Introduction

Corn stalk residues, including crop stalks, straw, husks, and other plant materials, are leftover materials from agricultural activities. Shredding these residues involves breaking them into smaller pieces, which offers benefits such as enhanced decomposition, improved soil health, easier handling and transportation, reduced fire risk, and the production of livestock feed. Various machines are designed to effectively chop corn stalk residues, including forage choppers, crop residue choppers, mulchers, and biofuel choppers. These machines vary in size, capacity, and efficiency and are tailored to handle different types of residues. Farm residues can also be processed using mechanical choppers, hand tools, grinders, or shredders. When selecting a chopping machine, it is important to consider factors such as capacity, power source, and maintenance requirements.

Cutting force and power consumption can be reduced in chopping corn stalk residues through various strategies and optimization techniques. By reducing the cutting force, more compact mechanical parts of the cutting equipment can be used, leading to greater energy savings [1]. Factors affecting cutting force and power consumption are: cutting speed, moisture content, cutting energy requirement, milling vibration and cutting tooth errors. Higher cutting speeds generally require more power and result in higher cutting forces. High moisture content can increase cutting force and power consumption. By reducing shearing force, optimizing cutting parameters, and using energy consumption modelling, energy savings can be achieved [2]. These approaches contribute to a more efficient and sustainable agricultural residue harvesting processes [3]. It is also important to consider a reduction in the size of small grain residues to promote uniform distribution without increasing energy costs [4]. Overall, optimizing cutting parameters can improve residue management, increase soil organic matter, prevent erosion, and increase agricultural yields [5].

### 1.1 Chopping technologies and conditions on maize processing

The use of different corn processing technologies (such as conventional rollers, and Shred ledge Crop Processors) affects the quality of chopped corn. Shred ledge processors achieved the highest degree of grain processing and tended to produce the highest amount of physiologically effective fiber, resulting in greater nutrient loss after rumen incubation [6]. Experimental studies were conducted to identify optimal cutting process parameters for corn stalks, which can simultaneously reduce cutting force and power consumption, leading to more efficient cutting processes [1,7]. The moisture level and machine speed are identified to produce good levels of cut length and length remaining in the soil during the cutting process [8]. A novel double rollers type stalk cutting and maintaining device is proposed and highlights the rotation speed and dynamic support affecting the cutting process and passage rate of corn stalks [9]. Cutting process significantly affects the quality and digestibility of maize silage [[10], [11], [12]]. Optimum conditions for cutting corn for silage production include consideration of cutting height, genetic material, and processing method, with shredder processors showing promising results in improving corn silage quality [13,14]. Adjusting planting density and nitrogen application rate also plays a critical role in optimizing the growth, yield and quality of silage maize [15]. A study on cutting corn stalks using water jet technology found that the cutoff ratio of stalks and stem nodes increased with water jet pressure and decreased with target distance and traverse speed [16]. Experimental studies on the cutting process of stalk crops revealed the influence of cutting speed, raw material supply and number of counter cuts on specific energy consumption and average particle size, providing insights into the optimization of cutting process parameters [17].

### 1.2 Research on corn stalk cutting and crushing

Corn stalk cutting and crushing research is still continuing both nationally and internationally with attention directed towards optimizing techniques of processing that will improve its efficiency and effectiveness in crop residues management.

#### 1.2.1 Domestic research

The research in India about corn stalks slicing and pulverizing is improving particularly in the context of agricultural mechanization and biomass utilization. It involves studies on the mechanical properties of corn stalks which are aimed at optimization of agricultural machinery design to enhance biomass processing efficiency. For instance, the radial compression and bending mechanical characteristics of corn stalk were investigated using a universal material testing machine, which contributes to corn crop growth and serves as a foundation for equipment development [18].

#### 1.2.2 International research

Internationally, corn stalk crushing mechanisms are also being researched more and more. For example, a research project that was published by the American Society of Agricultural and Biological Engineers resulted in a corn stalk crushing and throwing device. In this study, kinematics and dynamic balance analysis were employed to optimize the speed and configuration of the crushing blades thereby greatly enhancing the performance [19]. Additionally, Niu. et al., 2021 [19] examined various parameters such as cutter shaft speed, blade number, inclination angle resulting in improved theoretical length and quality of crushed stalks.

China has made great strides in developing sophisticated machinery and methods for processing corn stalks. An investigation at Xinjiang Academy of Agricultural Sciences on air flow pattern inside 9FF square bale cornstalk pulveriser showed that specific hammer racks, blades and sieves can make high speed crashing efficiency better [20]. Another research work aimed at developing discrete element model to simulate cutting-crushing process optimization for maize stalks. This model shows the mechanical characteristics and interactions in the process better, hence can be used to improve equipment design [21]. Moreover, an experimental investigation has been done on crushing behaviour of square-baled corn stalks and raised issues such as blade angle, moisture content, loading speed that impact significantly on the crush force and efficiency [22].

### 1.3 Outline of the present study

Motivated by the work of above researchers, this article deals with optimization of cutting processes for corn stalks. These are significant sources of biomass and forage. There is a need for minimizing both cutting force and power consumption. A modified Taguchi's method is used for optimization of the problem. Chauvenet's criterion is applied to ensure the statistical validity of scatter in repeated test data [1,7,23]. Results of analysis of variance (ANOVA) are presented to identify the optimal chopping process parameters. This study provides valuable insights for the efficient operation of chopping machines handling corn stalk residues.

## 2 Materials and methods

This section highlights earlier studies on corn stalks chopping process and its optimal parameters. The drawbacks in the previous studies and improvements in the current study are highlighted. A modified Taguchi method is adopted with a simple and reliable multi-objective optimization procedure [24] to reduce cutting force and power consumption in chopping corn stalk residues. It also presents the details on determination of statistically allowable limits applying the Chauvenet's criterion to the output responses from repeated tests [1], development of empirical relationships for the cutting force and cutting power in terms of chopping process parameters, and their validation.

### 2.1 Studies on corn stalks chopping process

**Figure-1:**
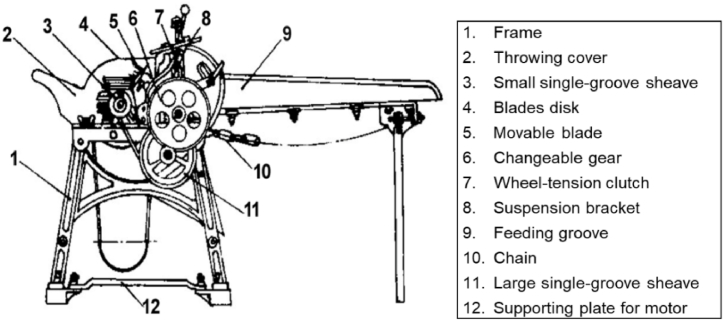
93 ZT-1000 straw chopper [25].

The setup was made based on the outline of a 93 ZP-1000 straw chopper produced by the Liaoning Fengcheng Donfeng Machinery Factory (Liaoning, Dandong, China). Fig. 1 illustrates a 3D model of the chopping device. Vu et al. [1] obtained maize stalks from Vietnam and stored in an air-conditioned room for one week after harvest. The wet based moisture content of the samples was 81 % then measured by using drying–weighting method. They employed a commercial chopper basing on the outline of 93 ZP-1000 straw chopper [25] (Figure-1) and adopted Taguchi's design of experiments in corn stalks chopping processes.

(**Source**: Food and Agriculture Organization of the United Nations, figure reproduced with permission.)

**Figure-2:**
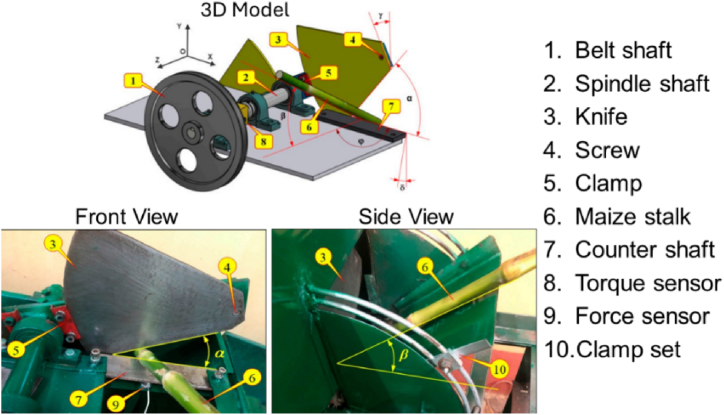
Experimental setup [1].

The experimental setup is illustrated in Fig. 2, where all parts are labelled similarly to those in Fig. 1. A commercial 1.5 kW DC motor, operating on a voltage source between 50 and 220 V, powered the setup. An alternating-current (AC) variable transformer was used to modify the voltage, thereby adjusting the speed of the cutting spindle. The approach angle (α) was controlled by the screw and clamp set (Fig. 2, parts 4 and 5), while the feed angle (β) was adjusted using the clamp set (Fig. 2, part 10). A dynamic force sensor (type 9712A500) was positioned beneath the counter shear bar to measure the cutting force in real time. Cutting force data was recorded using a DAQ (type NI-USB-600F8) and the NI Signal Express software. The average power consumption is the amount of energy consumed per unit time. Consequently, given a cutting force F_C_ and the corresponding cutting velocity V, the cutting power P_C_ can be expressed as:

(1) $$P_{C}=F_{C}\times V$$

(**Source:** MDPI journal, authors were permitted to reprint the figure)

### 2.2 Straw chopper mechanism

Agricultural chopping systems are generally designed using two main cutting principles: scissor shearing, which provides shear stress, and rotary knives, which generate both impact and shear stress on the stalks [26]. In the case of scissor shearing, the plants are pressed against a fixed counter edge with the aid of a cutter. The knife speed is divided into two components: chopping speed, which penetrates the material, and sliding cut speed [27].

Working of straw chopper, the 93 ZT-1000 straw chopper functions on cutting and grinding principles. It particularly processes residue of crops like maize stover, wheat and rice straw, bean vine and tuber vine. This is the simple working principle of 93 ZT-1000 straw chopper:

• **Feeding**: The machine receives the straw from a hopper. It has a controlled feeding rate to ensure that it operates smoothly and at maximum efficiency during processing. This is done in such a way that in the machine, the primary cutting mechanism does a first chop of the straw into smaller pieces. As such this reduces load on grinding chamber, hence increasing overall efficiency.
• **Grinding Chamber**: This part of machine is where rotating hammers further grind chopped pieces produced by the chopping process. The hammer slaps against high speed strike straw causing it to be impacted with serrated plates screens inside the chamber.
• **Separation and Collection**: At this point, grounded particles are screened out for separation where larger ones are recirculated for re-grinding while those that conform to desired size are collected through an outlet from which they exit.
• **Output**: Finally, fine particles consisting of straw that can be used as feed or soil conditioner form part of end products.

The 93 ZT 1000 straw chopper excels, in enhancing the taste and nutritional value of straw for animals making it a valuable feed option. With an emphasis, on efficiency and lasting build it guarantees reliable performance and increased productivity.

### 2.3 Experimentation for data acquisition

Experiments were designed by selecting three chopping process parameters: cutting velocity (V), approach angle (α), and feed angle (β), each at three levels. These levels are V = 4.4 m/s, 5.66 m/s, 6.91 m/s; α = {0°, 30°, 60°}; and β = {0°, 25°, 50°}. Tests were conducted following Taguchi's L9 orthogonal array (OA), with each set of chopping process parameters repeated three times. The two output responses in the optimization problem are the peak values of cutting force (F) and cutting power (P). A total of 27 tests were conducted. Chauvenet's criterion was applied to ensure the statistical validity of the test data [1,7,23]odified Taguchi method is adopted to develop empirical relations for the output responses (F,P) in terms of input variables (V, α, β) and to identify the optimal chopping process parameters.

## 3 Results and discussion

From the experimental test data, the sets of chopping process parameters according to Taguchi's L9 OA and the output responses are shown in Table 1. The numbers in the first column of Table 1 represent the set numbers for the chopping process parameters (V, α, β) according to the L9 OA in columns 2 to 4. The fifth column provides the test numbers for the three repeated tests in Ref. [1], with the output responses for the cutting force in columns 6 to 8 and the cutting power in columns 9 to 11. Therefore, for the nine sets of chopping parameters with three repeated tests each, 27 tests were conducted in total, which should not be confused with a full factorial design of experiments involving three factors (V, α, β) at three levels each.

**Table-1:** Sets of chopping process parameters (V, α, β) selected in Taguchi's L_9_ OA and the output responses (F and P).

Test run	Chopping process parameters			Test Number [1]	Cutting force, F (N)			Cutting power, P (W)		
	V (m/s)	α (^o^)	β (^o^)		Test 1	Test 2	Test 3	Test 1	Test 2	Test 3
1	4.4	0	0	1,2,3	706.07	617.68	502.10	82.38	72.06	58.58
2	4.4	30	25	4,5,6	313.99	264.13	276.61	36.63	30.82	32.27
3	4.4	60	50	7,8,9	261.94	269.8	263.62	30.56	31.48	26.32
4	5.66	0	25	10,11,12	485.10	479.44	414.88	72.77	71.92	62.23
5	5.66	30	50	13,14,15	312.80	255.07	242.62	46.93	38.26	36.39
6	5.66	60	0	16,17,18	287.93	267.58	261.97	43.19	40.13	39.28
7	6.91	0	50	19,20,21	344.59	317.39	289.11	63.17	58.19	52.99
8	6.91	30	0	22,23,24	307.19	273.2	250.51	56.32	50.09	45.93
9	6.91	60	25	25,26,27	385.38	361.59	317.42	70.65	66.29	58.19

For minimizing F and P, the S/N ratio transformation ("smaller-the-better") applied is: $S/N=-10\times \log\;10\left(\frac{1}{n}\sum_{i=1}^{n}\eta_{i}^{2}\right)$, where n is the number of replications and η_i_ is the value of the output response for i = 1, 2, …, n. Although n = 3 in this case, the authors in Ref. [1] incorrectly applied the S/N ratio transformation to the output response of each test (i.e., n = 1). This incorrect application involves additional computation and provides no advantage in seeking the optimal solution.

The smallest cutting power, P = 26.32 W, is observed in test number 9, which also has a higher cutting force, F = 263.6 N. Conversely, the smallest cutting force, F = 242.6 N, is found in test number 15, with a high cutting power, P = 36.39 W. Based on the Grey Relational Analysis (GRA) and Analysis of Variance (ANOVA) techniques, the optimal set of input parameters (V, α, β) = (4.4 m/s, 30°, 50°) yields a combination of both relatively small cutting force, F = 251.7 N, and cutting power, P = 26.2 W. For each set of chopping process parameters listed in Table-1, the values of F and P vary significantly across three repeated tests. Therefore, for a set of optimal chopping process parameters, repeated tests should provide the range of output responses.

Vu et al. [7] built the experimental plan as per the Central Composite Design (CCD) and used the response surface methodology (RSM) to obtain relationships for the cutting force (F) and the power consumption (P), as functions of chopping process parameters (V, α, β) as:

(2) $$F=411+151V-19.72\alpha -12.49\beta -19.72V^{2}+0.1263\alpha^{2}+0.0695\beta^{2}+1.196V\alpha +0.173V\beta +0.1080\alpha \beta$$

(3) $$P=-41+44.8V-2.54\alpha -1.595\beta -3.62V^{2}+0.01955\alpha^{2}+0.01131\beta^{2}+0.108V\alpha +0.0555V\beta +0.01598\alpha \beta$$

The optimal set of input parameters, (V, α, β) = (4.4 m/s, 40.2°, 35°) for a combination of both relatively small cutting force, F = 237.8 N and cutting power, P = 23.8 W obtained using equations (2), (3) by mean of the response optimiser function of Minitab R18 software. For the set of input parameters, (V, α, β) = (5.66 m/s, 30°, 25°), equation (2) gives F = 212.39N, while the test data in Ref. [7] from 6 repeated tests are varying from 230.14N to 367.25N. For this set of input parameters, equation (3) gives P = 43.36 W, while the test data in Ref. [7] from the 6 repeated tests are varying from 34.52 W to 55.09 W. Large deviation in F and P values observed from the test data to the empirical relationships (2) and (3). Hence, the range of output responses for a set of optimal chopping process parameters will be more appropriate.

Defining $X_{1}=\frac{V-5.66}{1.255}$; $X_{2}=\frac{\alpha -30}{30}$; and $X_{3}=\frac{\beta -25}{25}$, empirical relationships developed for F and P using RSM are [23]

(4) $$F=232.8-35X_{1}-103.4X_{2}+8.9X_{3}+17.1X_{1}^{2}+123.5X_{2}^{2}+28.4X_{3}^{2}+119.7X_{1}X_{2}-42.5X_{1}X_{3}$$

(5) $$P=38.79+6.71X_{1}-13.28X_{2}+1.63X_{3}+1.15X_{1}^{2}+16.89X_{2}^{2}+0.10X_{3}^{2}+14.78X_{1}X_{2}-5.66X_{1}X_{3}$$

**Figure-3:**
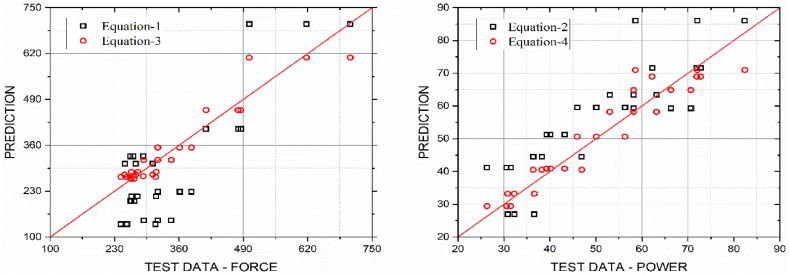
Comparison of cutting force (N) and cutting power (W) from empirical relationships (1) to (4) in Refs. [7,23] with test data [1] showing large deviation.

For the set of input parameters, (V, α, β) = (5.66 m/s, 30°, 25°), equation (4) gives F = 232.8 N, while the test data in Ref. [7] from 6 repeated tests are varying from 230.14 N to 367.25 N. For this set of input parameters, equation (5) gives P = 38.79 W, while the test data in Ref. [7] from the 6 repeated tests are varying from 34.52 W to 55.09 W. Large deviation in F and P values observed from the test data [7] to the empirical relationships (4) and (5) in Ref. [23]. Figure-3 shows large deviations in F and P values from equations (2), (3), (4), (5) to the 27 test data in Ref. [1].

### 3.1 Statistically allowable limits

As in Ref. [23], Chauvenet's criterion is applied to the output responses of the repeated tests in Table-1 and determined the statistically allowable limits to verify the acceptance of scatter in the repeated test data. The allowable limits for the output responses arrived as follows. For the 3 output responses ($\eta_{1},\eta_{2},\eta_{3})$, $\bar{\eta }=\frac{1}{3}\left(\eta_{1}+\eta_{2}+\eta_{3}\right)$, is the average value and $\eta_{\sigma }=\sqrt{\frac{1}{2}\left\{{\left(\eta_{1}-\bar{\eta }\right)}^{2}+{\left(\eta_{2}-\bar{\eta }\right)}^{2}+{\left(\eta_{3}-\bar{\eta }\right)}^{2}\right\}}$, is the standard deviation. The allowable lower limit, $\eta_{LL}=\bar{\eta }-1.38\times \eta_{\sigma }$. The allowable upper limit, $\eta_{UL}=\bar{\eta }+1.38\times \eta_{\sigma }$. Table-2 gives the statistically allowable limits for the repeated test data in Table-1. The scatter in 3 output responses for each test run in Table-1 is high. However, the output responses are within the statistically allowable limits. Varalakshmi et al. [23] used average values of F and P. They followed the modified Taguchi approach [28]. Due to large scatter in the test data, they were unable to match the test data within the estimated range. In such situations, it is more appropriate to consider the lower and upper limits of the output responses in the statistical analysis.

**Table-2:** Allowable limits of the output responses (F and P) for the data in Table-1.

Test run	Set of chopping process parameters			Cutting force (N)		Cutting power (W)	
	V (m/s)	α (^o^)	β (^o^)	$F_{LL}$	$F_{UL}$	$P_{LL}$	$P_{UL}$
1	4.4	0	0	476.46	749.77	54.54	87.47
2	4.4	30	25	249.10	320.71	29.06	37.42
3	4.4	60	50	259.40	270.83	25.66	33.25
4	5.66	0	25	405.93	513.66	60.89	77.05
5	5.66	30	50	218.44	321.91	32.77	48.29
6	5.66	60	0	253.53	291.36	38.03	43.71
7	6.91	0	50	278.70	355.33	51.09	65.14
8	6.91	30	0	237.62	316.33	43.56	57.99
9	6.91	60	25	307.17	402.40	56.32	73.77

Following Ross [29], ANOVA performed on the output responses of Table-2 and presented the results in Table-3. The mean values of ANOVA will be useful in estimating F and P using the additive law for any set of ($V_{i},\alpha_{j},\beta_{k}$) in which the subscripts (i,j,k) are 1–3 levels. (V_1_,V_2_,V_3_) = (4.4 m/s,5.66 m/s,6.91 m/s); (α_1_,α_2,_α_3_) = (0°,30°,60°); and $\left(\beta_{1},\beta_{2},\beta_{3}\right)=\left(0,25,50^{o}\right)$. Let ψ be the output response (F or P) and $\overset{˘}{\psi }$, is its estimate for ($V_{i},\alpha_{j},\beta_{k}$) $\psi_{mean}$, is the grand mean of the 9 test runs. $\psi \left(V_{i}\right)$, $\psi \left(\alpha_{j}\right)$ and $\psi \left(\beta_{k}\right)$ are designated as mean values of ψ to the $i^{th}$ level of V, $j^{th}$ level of α, and $k^{th}$ level of β.

**Table-3:** ANOVA and %contribution of the chopping process parameters.

Parameters	1st mean	2nd mean	3rd mean	%Contribution
Lower limit of $F_{LL}$ (N): grand mean = 298.48 N				
V	328.32	292.63	274.50	7.5
Α	387.03	235.05	273.37	62.7
Β	322.54	320.73	252.18	16.1
Upper limit of $F_{UL}$ (N): grand mean = 393.59 N				
V	447.10	375.64	358.02	7.2
Α	539.59	319.65	321.53	51.9
Β	452.49	412.26	316.02	16.0
Lower limit of $P_{LL}$ (W): grand mean = 43.55 W				
V	36.42	43.90	50.32	22.0
Α	55.51	35.13	40.00	51.5
Β	45.38	48.76	36.50	18.2
Upper limit of $P_{UL}$ (W): grand mean = 58.23 W				
V	52.71	56.35	65.64	9.3
Α	76.56	47.90	50.24	53.0
Β	63.06	62.75	48.89	13.7

Following the additive law [9], estimate $\overset{˘}{\psi }$ for the specified ($V_{i},\alpha_{j},\beta_{k}$) is

(6) $${\hat{\psi }}_{ijk}=\psi \left(V_{i},\alpha_{j},\beta_{k}\right)=\psi \left(V_{i}\right)+\psi \left(\alpha_{j}\right)+\psi \left(\beta_{k}\right)-2\times \psi_{mean}$$

From the deviations of the test data and the estimate, the corrections ($\Delta \psi_{LL}$ and $\Delta \psi_{UL}$) for the lower and upper limits of ψ are

(7) $$\Delta \psi_{LL}=\min\left\{\psi_{111}-{\hat{\psi }}_{111},\psi_{122}-{\hat{\psi }}_{122},\psi_{133}-{\hat{\psi }}_{133}\right\}$$

(8) $$\Delta \psi_{UL}=\max\left\{\psi_{111}-{\hat{\psi }}_{111},\psi_{122}-{\hat{\psi }}_{122},\psi_{133}-{\hat{\psi }}_{133}\right\}$$

For the case, $\psi =F_{LL}$, the estimates in (7) obtained from (6) using Table-3 are

$${\hat{\psi }}_{111}=328.32+387.03+322.54-2\times 298.48=440.93$$

$${\hat{\psi }}_{122}=328.32+235.05+320.73-2\times 298.48=287.14$$

$${\hat{\psi }}_{133}=328.32+273.37+252.18-2\times 298.48=256.91$$

The test data $\psi_{111},\psi_{122}\text{,}$ and $\psi_{133}$ in (7) obtained from Table-2 for the case $\psi =F_{LL}$ are

$$\psi_{111}=476.46$$

$$\psi_{122}=249.10$$

$$\psi_{133}=259.40$$

The correction for the lower limit of ψ from (7) is

$$\Delta \psi_{LL}=\min\left\{476.46-440.93,249.10-287.14,259.40-256.91\right\}=\min\left\{35.53,-38.04,2.49\right\}=-38.04$$

For the case, $\psi =F_{UL}$, the estimates in (7) obtained from (6) using Table-3 are

$${\hat{\psi }}_{111}=447.10+539.59+452.49-2\times 393.59=652.0$$

$${\hat{\psi }}_{122}=447.10+319.65+412.26-2\times 393.59=391.83$$

$${\hat{\psi }}_{133}=447.10+321.53+318.02-2\times 393.59=299.47$$

The test data $\psi_{111},\psi_{122}\text{,}$ and $\psi_{133}$ in (7) obtained from Table-2 for the case $\psi =F_{UL}$ are

$$\psi_{111}=749.77$$

$$\psi_{122}=320.71$$

$$\psi_{133}=270.83$$

The correction for the upper limit of ψ from (7) is

$$\Delta \psi_{UL}=\max\left\{749.77-652.0,320.71-391.83,270.83-299.47\right\}=\max\left\{97.77,-71.12,-28.64\right\}=97.77$$

For test run-1, equation (6) gives the estimate of $F_{LL}=440.93$. The correction to this estimate is: $\Delta F_{LL}=-38.04$. The expected result should be above or equal to the value of $F_{LL}+\Delta F_{LL}=440.93-38.04=402.89$, which confirms the data of 476.46 in Table-3. Similarly, for test run-1, estimate of $F_{UL}=652.0$ from (6) and the correction to this estimate, $\Delta F_{UL}=97.77$. The expected result should be below or equal to the value of $F_{UL}+\Delta F_{UL}=652.0+97.77=749.77$, which confirms the data of 749.77 in Table-3. From the above calculations, the expected range of cutting force (F) for test run-1 is from 402.89 to 749.77 N. The data in Table-1 from 3 repeated tests are 706.07, 617.68 and 502.1N, which fall within the expected range (see Table-4 for other test runs).

**Table-4:** Comparison of F and P estimates with test data [1].

Test run	Repeated test data [1]			Additive law (Eq. (6))		Estimated range	
	Test-1	Test-2	Test-3	Lower level	Upper level	from	to
Cutting force, F (N): (Corrections, $\Delta F_{\text{LL}}=-38.04\;N$ and $\Delta F_{\text{UL}}=97.77$ N)							
1	706.07	617.68	502.1	440.92	652.00	402.88	749.77
2	313.99	264.13	276.6	287.14	391.83	249.10	489.60
3	261.94	269.8	263.6	256.90	297.48	218.86	395.25
4	485.1	479.44	414.8	403.43	540.31	365.39	638.08
5	312.8	255.07	242.6	182.90	224.14	144.86	321.91
6	287.93	267.58	261.9	291.57	362.48	253.53	460.25
7	344.59	317.39	289.1	316.74	426.45	278.70	524.22
8	307.19	273.2	250.5	235.12	342.98	197.08	440.75
9	385.38	361.59	317.4	271.63	304.63	235.59	402.40
Cutting power, P (W): (Corrections, $\Delta P_{\text{LL}}=-4.15\;W$ and $\Delta P_{\text{UL}}=11.61\;W$)							
1	82.38	72.06	58.58	50.21	75.86	46.06	86.87
2	36.63	30.82	32.27	33.21	46.90	29.06	57.97
3	30.56	31.48	26.32	25.84	35.39	21.69	46.40
4	72.77	71.92	62.23	61.07	79.19	56.97	90.20
5	46.93	38.26	36.39	28.44	36.68	24.29	47.69
6	43.19	40.13	39.28	42.18	53.19	38.03	64.20
7	63.17	58.19	52.99	55.24	74.62	51.09	85.63
8	56.32	50.09	45.93	43.74	60.13	39.59	71.14
9	70.65	66.29	58.19	51.99	62.16	47.84	73.17

**Table-5:** Cutting force estimates comparison with test data of central composite design [7].

Test sequence Number	Set of chopping process parameters			Cutting force, F (N)				
				Test [7]	Eq. (2) [7]	Eq. (4) [23]	$F_{LL}$ (Eq. (9))	$F_{UL}$ (Eq. (10))
	V (m/s)	α (^o^)	β (^o^)					
1	4.4	0	0	706.07	703.69	608.50	402.87	749.77
2	6.91	0	0	548.56	537.65	384.72	349.12	660.62
3	4.4	60	0	282.26	290.91	162.30	289.21	531.71
4	6.91	60	0	325.32	304.99	415.42	235.46	442.56
5	4.4	0	50	392.18	291.00	711.30	332.51	613.31
6	6.91	0	50	344.59	146.67	318.19	278.76	524.16
7	4.4	60	50	312.86	202.22	265.10	218.85	395.25
8	6.91	60	50	425.04	238.01	348.89	165.10	306.10
9	4.4	30	25	313.99	214.85	284.90	249.09	489.60
10	6.91	30	25	210.87	149.72	214.91	195.34	400.45
11	5.66	0	25	485.10	406.24	459.70	365.39	638.08
12	5.66	60	25	327.59	245.89	252.90	251.73	420.02
13	5.66	30	0	359.39	375.73	252.30	215.21	458.37
14	5.66	30	50	312.86	135.94	270.10	144.85	321.91
15	5.66	30	25	367.25	212.39	232.80	213.41	418.14
16	5.66	30	25	364.98	212.39	232.80	213.41	418.14
17	5.66	30	25	293.59	212.39	232.80	213.41	418.14
18	5.66	30	25	243.74	212.39	232.80	213.41	418.14
19	5.66	30	25	236.94	212.39	232.80	213.41	418.14
20	5.66	30	25	230.14	212.39	232.80	213.41	418.14

### 3.2 Refined empirical relationships

As in Refs. [24,30,31], the levels of the chopping process parameters (V,α,β) are transformed from −1 to 1, and represented the mean values of output responses $\left(F_{LL},F_{UL},P_{LL},P_{UL}\right)$ in ANOVA Table-3 for each parameter in quadratic polynomial and adopting the additive law (Eq. (5)), the following empirical relationships with corrections ($\Delta F_{LL}=-38.04$, $\Delta F_{UL}=97.77\;N$, $\Delta P_{LL}=-4.15\;W$ and $\Delta P_{UL}=11.61\;W$) are developed.

(9) $$F_{LL}=213.41-26.91\xi_{1}+8.77\xi_{1}^{2}-56.83\xi_{2}+95.15\xi_{2}^{2}-35.18\xi_{3}-33.38\xi_{3}^{2}$$

(10) $$F_{UL}=418.14-44.54\xi_{1}+26.92\xi_{1}^{2}-109.03\xi_{2}+110.91\xi_{2}^{2}-68.23\xi_{3}-28\xi_{3}^{2}$$

(11) $$P_{LL}=36.54+6.95\xi_{1}-0.53\xi_{1}^{2}-7.75\xi_{2}+12.63\xi_{2}^{2}-4.44\xi_{3}-7.82\xi_{3}^{2}$$

(12) $$P_{UL}=62.14+6.46\xi_{1}+2.82\xi_{1}^{2}-13.16\xi_{2}+15.5\xi_{2}^{2}-7.08\xi_{3}-6.77\xi_{3}^{2}$$

Here, the chopping process parameters, namely, the cutting velocity (V) is represented by $\xi_{1}=\frac{\left(V-5.66\right)}{1.26}$; the approach angle (α) is represented by $\xi_{2}=\frac{\left(\alpha -30\right)}{30}$; and the feed angle is represented by $\xi_{3}=\frac{\left(\beta -25\right)}{25}$.

**Figure-4:**
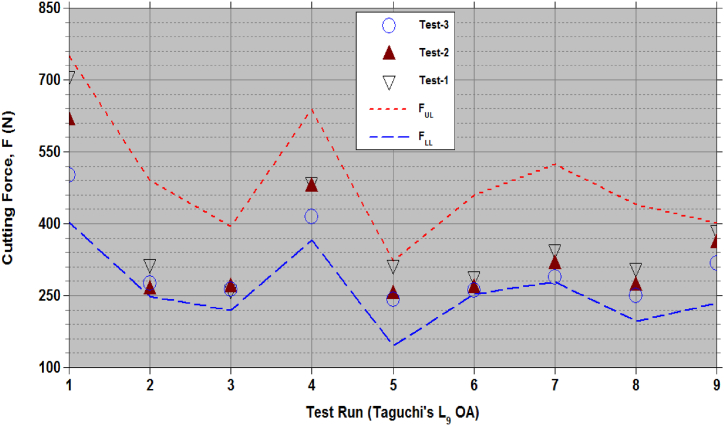
Cutting force (F) with the sets of chopping process parameters as per the test runs in the Taguchi's L_9_ OA.

**Figure-5:**
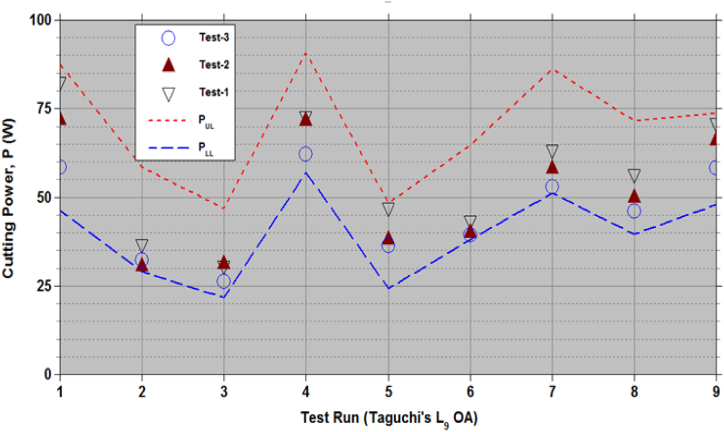
Cutting power (P) with the sets of chopping process parameters as per the test runs in the Taguchi's L_9_ OA.

Figure-4, Figure-5 show the cutting force (F) and power (P) generated using empirical relationships (9) to (12) for the sets of (V,α,β) as per Taguchi's L_9_ OA test runs. Data [1] from 3 repeated tests in Figure-4, Figure-5 are within the lower and upper limits. Table-5, Table-6 presents the CCD test data of F and P [7] and estimates from empirical relationships (8) to (11). For test sequence numbers from 15 to 20 in Table-5, Table-6, the set of process variables, (V, α, β) = (5.66 m/s, 30°, 25°). Equations (9), (10) give the limits of the cutting force, $F_{LL}$ = 213.41N and $F_{UL}$ = 418.14N, while the test data in Ref. [7] from 6 repeated tests vary from 230.14N to 367.25N. Equations (11), (12) gives the limits of the cutting power, $P_{LL}$ = 36.54 W and $P_{UL}$ = 62.14 W, while the test data in Ref. [7] from 6 repeated tests vary from 34.52 W to 55.09 W. These observations confirm the validity of the developed empirical relationships (9) to (12). Test sequence number 8, the test data is outside the estimated limits. The set of process variables corresponds to this 8th test sequence (6.91 m/s, 60°, 50°). Repeated tests were not conducted in Ref. [7] for this case to verify the reported data falls under outlier or not as per the Chauvenet's criterion. Other than the data for this set of process variables, all test data fall within the acceptable limits.

### 3.3 multi-objective optimization

In corn stalks cutting process, the cutting force minimization is important for the energy efficiency and compact mechanical design of the chopping device. Use of high cutting velocities consumes considerable power even with low shear forces. Hence, it is necessary to reduce the cutting force and power consumption.

From ANOVA Table-3, a set of chopping process parameters $V_{3}\alpha_{2}\beta_{3}$ is found for minimum cutting force ($F_{LL}orF_{UL}$), while another set $V_{2}\alpha_{2}\beta_{3}$ for minimum cutting power ($P_{LL}orP_{UL}$). Here subscripts denote the level of parameter. These sets are different for minimum F and P. A multi-objective optimization technique is employed to identify a set of optimal process parameters as in Refs. [[32], [33], [34]]. This technique involves constructing a multi-objective function (ζ), which considers force ($F_{LL}$) and power ($P_{LL}$). These parameters are normalized in ANOVA Table-3 are normalized considering maximum values of ${\left(F_{LL}\right)}_{\max}$ = 476.46 N; and ${\left(P_{LL}\right)}_{\max}$ = 60.89 W in Table-2. Introducing the positive weighing factors $\omega_{1}$ and $\omega_{2}$ (which satisfy $\omega_{1}+\omega_{2}=1$), a single objective function (ζ) is constructed in the form

(13) $$\zeta =\omega_{1}\left(\frac{F_{LL}}{{\left(F_{LL}\right)}_{\max}}\right)+\omega_{2}\left(\frac{P_{LL}}{{\left(P_{LL}\right)}_{\max}}\right)$$

Minimization of ζ provides minimum $F_{LL}$ and $P_{LL}$ with a set of optimal chopping parameters. For $\omega_{1}$ = 1($\Rightarrow \omega_{2}$ = 0), ζ yields minimum $F_{LL}$, while for $\omega_{2}$ = 1($\Rightarrow \omega_{1}$ = 0), ζ yields minimum $P_{LL}$. Both $\omega_{1}$ and $\omega_{2}$ are considered to obtain a set of optimal process parameters. Mean values of $F_{LL}$ and $P_{LL}$ in ANOVA Table-3 are normalized by ${\left(F_{LL}\right)}_{\max}$ = 476.46 N; and ${\left(P_{LL}\right)}_{\max}$ = 60.89 W respectively. ANOVA Table-7 for ζ is generated using (5) and specifying $\omega_{1}$ and $\omega_{2}$. Table-7 also presents a set of optimal parameters to each case study. Table-8 gives optimal chopping parameters for specific conditions along with F and P estimates. Test data [1] for the optimal solution falls within the expected range.

**Table-6:** Cutting power estimates comparison with test data of central composite design [6].

Test sequence Number	Set of chopping process parameters			Cutting power, P (W)				
				Test [7]	Eq. (3) [7]	Eq. (5) [23]	$P_{LL}$ (Eq. (11))	$P_{UL}$ (Eq. (12))
	V (m/s)	α (^o^)	β (^o^)					
1	4.4	0	0	82.37	86.04	70.99	46.14	87.46
2	6.91	0	0	100.57	95.72	66.17	59.99	100.28
3	4.4	60	0	32.93	32.53	14.87	30.64	61.14
4	6.91	60	0	59.64	58.48	68.94	44.49	73.96
5	4.4	0	50	45.75	46.77	85.57	37.26	73.30
6	6.91	0	50	63.17	63.42	58.20	51.11	86.12
7	4.4	60	50	36.5	41.20	29.45	21.76	46.98
8	6.91	60	50	77.92	74.12	60.97	35.61	59.80
9	4.4	30	25	36.63	26.97	33.23	29.09	58.50
10	6.91	30	25	38.66	48.27	46.58	42.94	71.32
11	5.66	0	25	72.77	71.65	68.96	56.97	90.80
12	5.66	60	25	49.14	50.27	42.40	41.47	64.48
13	5.66	30	0	53.91	56.33	37.26	33.16	62.44
14	5.66	30	50	46.93	44.53	40.52	24.28	48.28
15	5.66	30	25	55.09	43.36	38.79	36.54	62.14
16	5.66	30	25	54.75	43.36	38.79	36.54	62.14
17	5.66	30	25	44.04	43.36	38.79	36.54	62.14
18	5.66	30	25	36.56	43.36	38.79	36.54	62.14
19	5.66	30	25	35.54	43.36	38.79	36.54	62.14
20	5.66	30	25	34.52	43.36	38.79	36.54	62.14

**Table-7:** ANOVA on ζ with different $\omega_{1}$ and $\omega_{2}$.

Chopping Parameters	1st Mean	2nd Mean	3rd Mean	Optimal Solution
${\left(F_{\text{LL}}\right)}_{\min}$: $\omega_{1}=1\text{and}\omega_{2}=0$				
V	0.6891	0.6142	0.5761	$V_{3}\alpha_{2}\beta_{3}$
α	0.8123	0.4933	0.5737	
β	0.6769	0.6732	0.5293	
${\left(P_{\text{LL}}\right)}_{\min}$: $\omega_{1}=0\text{and}\omega_{2}=1$				
V	0.5981	0.7209	0.8265	$V_{2}\alpha_{2}\beta_{3}$
α	0.9116	0.5769	0.6570	
β	0.7452	0.8007	0.5996	
${\left(F_{\text{LL}}\right)}_{\min}$ and ${\left(P_{\text{LL}}\right)}_{\min}$: $\omega_{1}=\frac{1}{2}\text{and}\omega_{2}=\frac{1}{2}$				
V	0.6436	0.6676	0.7013	$V_{1}\alpha_{2}\beta_{3}$
α	0.8620	0.5351	0.6154	
β	0.7111	0.7370	0.5645

**Table-8:** Chopping parameters and estimates of F and P for specific conditions.

Chopping process parameters			Cutting force (N)		Cutting power (W)	
V (m/s)	α (^o^)	β (^o^)	$F_{LL}$	$F_{UL}$	$P_{LL}$	$P_{UL}$
${\left(F_{LL}\right)}_{\min}$: optimal set ($V_{3}\alpha_{2}\beta_{3}$)						
6.91	30	50	126.72	304.29	30.72	56.97
${\left(P_{LL}\right)}_{\min}$:optimal set ($V_{2}\alpha_{2}\beta_{3}$)						
5.66	30	50	144.86	321.91	24.29	47.69
			242.6–312.8^+^		*36.39–46.93^+^	
${\left(F_{LL}\right)}_{\min}$ and ${\left(P_{LL}\right)}_{\min}$: optimal set ($V_{1}\alpha_{2}\beta_{3}$)						
4.4	30	50	180.55	393.37	16.81	44.05
			251.7^+^		26.2^+^	

+ Test data [1].

Vu et al. [1] generated data by specifying 9 sets of chopping process parameters as per Taguchi's L9 OA. For each set they conducted chopping process 3 times. A total of 27 data was generated for output responses. The data identified a set of process parameter (4.4 m/s, 60°, 50°) for minimum consumption of power, P = 26.32 W while a set of process parameters (5.56 m/s, 30°, 50°) for minimum force, F = 242.60 N. These two minimum P and F values were lowest among the 3 repeated test data. While refined empirical relations provides the estimated force from 144.85 m/s to 321.91 m/s, whereas power varies from 21.76 W to 46.98 W. Minimum F and P obtained at different sets of process parameters. Hence Vu et al. [1] adapted Taguchi grey relation analysis and obtained optimum parameters (4.4 m/s, 30°, 50°) for which they conducted a single test and reported force = 251.70 m/s and power = 26.20 W. The refined empirical relationships for this optimum set of process parameters provide the cutting force from 180.55 m/s to 393.37 m/s, and cutting power from 16.81 W to 44.05 W. This confirms the validity of the refined empirical relationships.

**Figure-6:**
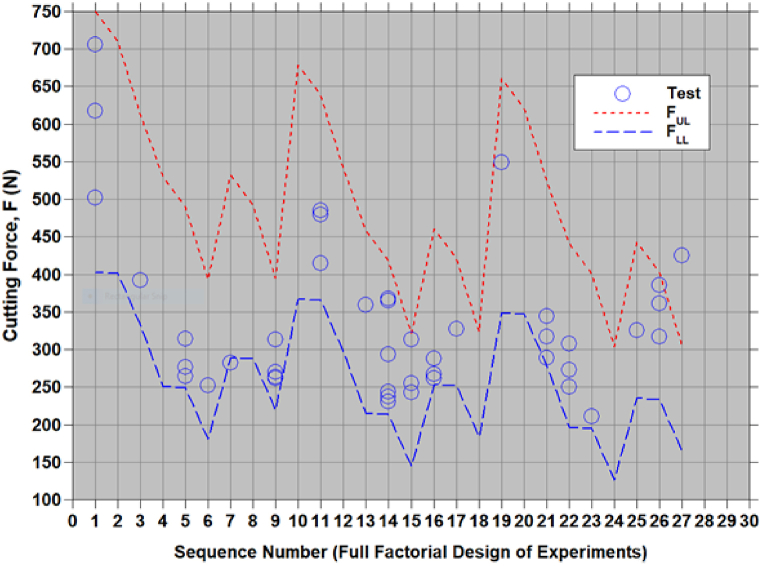
Comparison of estimates of cutting force using (8), (9) with test data [1,7].

**Figure-7:**
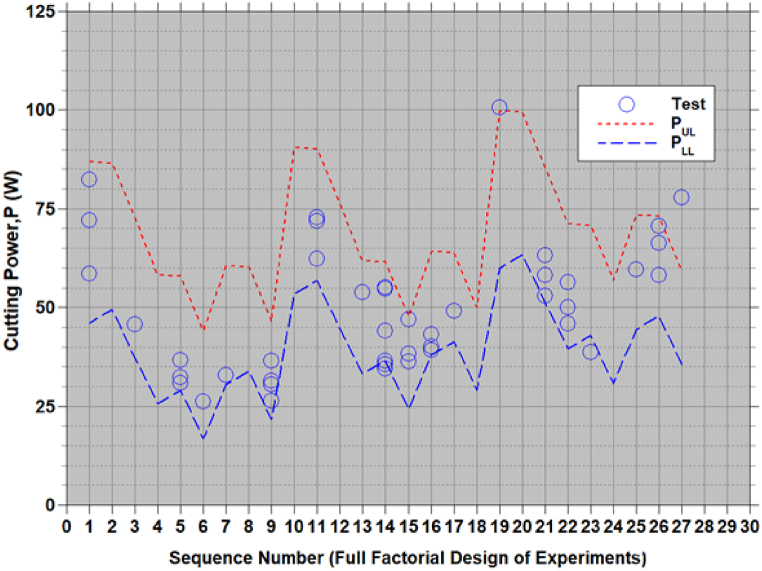
Comparison of estimates of cutting power using (10), (11) with test data [1,7].

A modified Taguchi method combined with a simple and reliable multi-objective optimization is followed to reduce cutting force and power consumption (F and P) in chopping corn stalk residues. Empirical relationships for cutting force and cutting power are developed in terms of chopping process parameters (viz., cutting velocity (V), approach angle (α) and feed angle (β)). The 3 levels assigned to each parameter are: V={4.4,5.66,6.91m/s}; $\alpha =\left\{0,30,60^{o}\right\}$; and $\beta =\left\{0,25,50^{o}\right\}$. For 3 parameters (V,α,β) with 3 levels, 27 sets of parameters $\left(\left(\left(\left(V_{i},\alpha_{j},\beta_{k}\right),k=1,2,3\right),j=1,2,3\right),i=1,2,3\right)$ can be constructed to generate the cutting force and power using equations (9), (10), (11), (12). These sets represents all 27 possible combinations of input variables (full factorial design of experiment) useful to generate the output responses (F & P). For each set of process parameter changes in F and P are shown in Figure-6, Figure-7 by equations (8), (9), (10), (11)). The scatter is reported test data [1,7] corresponding to the sets of input variables are also shown in Figure-6, Figure-7. These test data falls within the lower and upper bound of F and P. Increasing and decreasing trend of F and P, in Figure-6, Figure-7 are due to change in set of parameters which represented in terms of sequence numbers.

**Figure-8:**
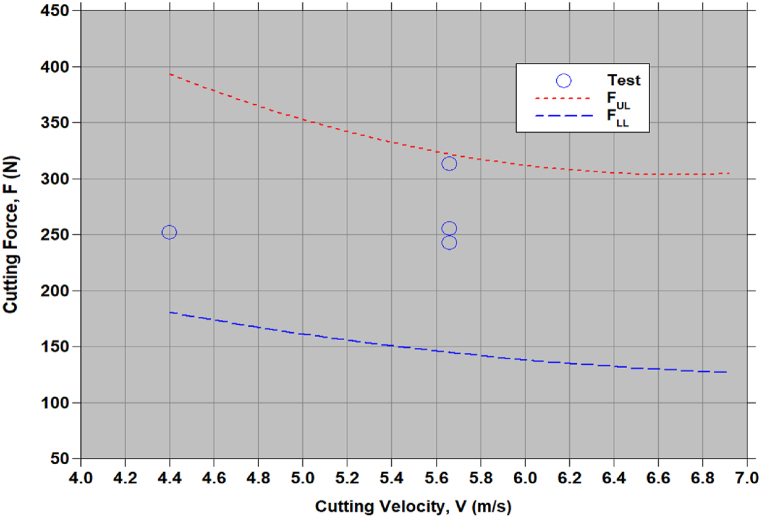
Variation of cutting force (F) with cutting velocity (V) for the optimal approach angle, $\alpha_{2}=$ 30° and the feed angle, $\beta_{3}=$ 50°.

**Figure-9:**
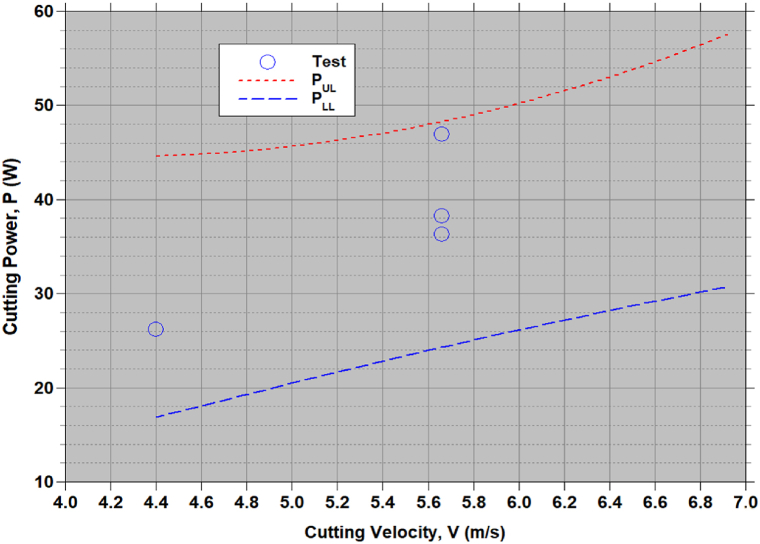
Variation of cutting power (P) with cutting velocity (V) for the optimal approach angle, $\alpha_{2}=$ 30° and the feed angle, $\beta_{3}=$ 50°.

A simple multi-objective optimization procedure is followed and obtained a set of optimal chopping process parameters, $\left(V_{1},\alpha_{2},\beta_{3}\right)=$ (4.4 m/s, 30°, 50°) for minimum F and P. To see trend of F and P with V for α, β can be seen in Figure-8, Figure-9. F is decreases and P increases with increasing in V. Fig. 8 shows slight decrease in the V, whereas in Fig. 9 shows the appraisable increase in P. As the required minimum F and P Fig. 9 shows 4.4 m/s is optimal. This confirm the identified optimal set of process parameter. The test data [1,7] fall within estimated lower and upper limits of F and P.

The refined empirical relationships equations (9), (10), (11), (12)) were developed using the experimental data from 9 sets of chopping process parameters as per Taguchi's L9 OA. These equations were validated with experimental data [1,7]. Figure-6, Figure-7 show the 27 test data of Taguchi's L9 OA [1] and 20 CCD test data [7] where the data falls within the estimated bounds. It should be noted that the wet based moisture content of the samples was 81 % then measured by using drying–weighting method. The large scatter observed in the repeated test data may be due to variations in the maize stock thickness. However, the test data fall within the limits of the refined empirical relationship.

## 4 Conclusions

This study conducts a comprehensive investigation into optimizing the chopping processes of corn stalks. To improve energy efficiency, cutting force and power consumption are simultaneously reduced by combining the modified Taguchi method with the concept of multi-objective optimization. Chauvenet's criterion is applied to identify outliers (if any) from repeated test data. Acceptances of repeated test data was confirmed with 95 % confidence by applying Chauvenet's criterion which is based on normal distribution concept. Using analysis of variance (ANOVA), the optimal chopping process parameters were identified. The following were drawn conclusions;

• Refined empirical relationships are developed for the cutting force and cutting power concerning the chopping process parameters (viz., cutting velocity, approach angle, and feed angle). These relationships are validated with test data.
• The optimal chopping process parameters were identified as velocity of 4.4 m/s, approach angle of 30°, and feeding angle of 50°. The refined empirical relationships indicated that the cutting force ranges from 180.55 N to 393.37 N, while the cutting power ranges from 16.81 W to 44.05 W.
• The large scatter in the repeated test data might be due to variability in the thickness of the corn stalk. Samples having 81 % moisture content.
• A multi-objective optimization technique is used to select a set of optimal process parameters, and the results are validated with test data. This study will be useful for the efficient operation of chopping machines.

All developed empirical relations are valid within the specified limits of the input variable. The methos of approach can be utilised in different agricultural residues and manufacturing processes. Furthermore, the influence of the corn stalk moisture content needs to be studied. Limitations of the present study include the residue moisture content and steam thickness.

## Funding

This research did not receive any specific grant from funding agencies in the public, commercial, or not-for-profit sectors.

## Data availability statement

All the relevant data are included in the manuscript. No separate repository is attached. There is no additional data available for this study.

## CRediT authorship contribution statement

**Kurre Prasanth Kumar Reddy:** Writing – review & editing, Writing – original draft, Validation, Software, Resources, Investigation, Formal analysis, Data curation, Conceptualization. **S. Ramesh Kumar:** Writing – review & editing, Supervision, Formal analysis. **Boggarapu Nageswara Rao:** Writing – review & editing, Writing – original draft, Supervision, Formal analysis, Data curation, Conceptualization.

## Declaration of generative AI and AI-assisted technologies in the writing process

During the preparation of this work the authors used Grammarly to verify grammatical correctness, enhance readability, and improve language. After using these tools, the authors reviewed and edited the content as needed and takes full responsibility for the content of the publication.

## Declaration of competing interest

The authors declare that they have no known competing financial interests or personal relationships that could have appeared to influence the work reported in this paper.
